# Responses to Drought Stress Modulate the Susceptibility to *Plasmopara viticola* in *Vitis vinifera* Self-Rooted Cuttings

**DOI:** 10.3390/plants10020273

**Published:** 2021-01-30

**Authors:** Lisa Heyman, Antonios Chrysargyris, Kristof Demeestere, Nikolaos Tzortzakis, Monica Höfte

**Affiliations:** 1 Department of Plants and Crops, Faculty of Bioscience Engineering, Ghent University, 9000 Ghent, Belgium; lisa.heyman@ugent.be; 2 Department of Agricultural Sciences, Biotechnology and Food Science, Cyprus University of Technology, 3036 Limassol, Cyprus; a.chrysargyris@cut.ac.cy (A.C.); nikolaos.tzortzakis@cut.ac.cy (N.T.); 3 Department of Green Chemistry and Technology, Faculty of Bioscience Engineering, Ghent University, 9000 Ghent, Belgium; kristof.demeestere@ugent.be

**Keywords:** climate change, irrigation, abiotic stress, biotic stress, grapevine, downy mildew, phytohormones, oxidative stress, abscisic acid

## Abstract

Climate change will increase the occurrence of plants being simultaneously subjected to drought and pathogen stress. Drought can alter the way in which plants respond to pathogens. This research addresses how grapevine responds to the concurrent challenge of drought stress and *Plasmopara viticola*, the causal agent of downy mildew, and how one stress affects the other. Self-rooted cuttings of the drought-tolerant grapevine cultivar Xynisteri and the drought-sensitive cultivar Chardonnay were exposed to full or deficit irrigation (40% of full irrigation) and artificially inoculated with *P. viticola* in vitro or in planta. Leaves were sampled at an early infection stage to determine the influence of the single and combined stresses on oxidative parameters, chlorophyll, and phytohormones. Under full irrigation, Xynisteri was more susceptible to *P. viticola* than the drought-sensitive cultivar Chardonnay. Drought stress increased the susceptibility of grapevine leaves inoculated in vitro, but both cultivars showed resistance against *P. viticola* when inoculated in planta. Abscisic acid, rather than jasmonic acid and salicylic acid, seemed to play a prominent role in this resistance. The irrigation-dependent susceptibility observed in this study indicates that the practices used to mitigate the effects of climate change may have a profound impact on plant pathogens.

## 1. Introduction

Climate change leads to rising temperatures, shifting precipitation patterns, and an increase in the frequency of extreme weather events. In particular, prolonged drought is putting a significant strain on many ecosystems [1]. Viticulture, notorious for its sensitivity to climate, is drastically affected by climate change [2,3]. With a yearly production of 77 million tonnes of grapes on 7 million ha [4], grapevine is one of the most important fruit crops worldwide. Many viticultural areas are already suffering from seasonal drought, and water availability may become the bottleneck of wine production. The Mediterranean, a hotspot for wine production, is one of the world’s regions that is most vulnerable to the impact of climate change [5]. Global warming is very likely to aggravate soil dryness in this region [5].

In the Eastern Mediterranean, Cyprus is known for its hot and arid summers and annual precipitation is projected to further decline by more than 20% by the middle of the century [6]. Viticulture has played an important role in Cyprus for over 5500 years [7]. Currently, vineyards cover about 5% of the agricultural area [8], but only 11% of the vine area is irrigated [8]. Most indigenous grapevine cultivars can be grown without irrigation since drought tolerance has been an important selection criterion. Since the late 1980s, however, international market demands have pushed the use of commercial cultivars in Cyprus. These introduced cultivars require more water and fertilizers because they are not adapted to the less fertile soils and the arid conditions in Cyprus [9].

Climate change also significantly affects the distribution, virulence, abundance, and host range of plant pathogens [10,11]. This will increase the likelihood of combined occurrence of drought and pathogen stress, which is already a very common event [12]. The complexity of the interactions between plants and pathogens gains another dimension with exposure to drought stress, resulting in a new state of stress [13]. Because of the overlap and crosstalk between biotic and abiotic stress responses, plants must produce a tailored response to specific stress combinations [14]. Thus, the response to the concurrent challenge cannot simply be interpolated from the independent stress response [12,14,15,16,17,18].

The net outcome of the host–pathogen interaction under drought conditions is dependent on plant genotype, the nature of the pathogens, and the timing, severity, and duration of the stress [19,20,21,22]. Concurrent drought stress most often aggravates disease [23,24], but it can also trigger resistance [25,26]. There has been much research on drought stress [27,28] and pathogen stress [29] in grapevine, but there is almost no knowledge about the simultaneous occurrence of drought and pathogen stress. Choi et al. [30] found a strong interaction between drought and Pierce’s disease (*Xylella fastidiosa*). A few other studies provide information about consecutive, rather than simultaneous, drought and pathogen stress after inoculation of intact plants with *Plasmopara viticola* [31] or of detached leaves with *Botrytis cinerea* [32].

The current study aimed to gain more insight into the response of grapevine to the single and combined occurrence of drought and biotic stress and the influence of their interplay on disease susceptibility. For this purpose, an introduced grapevine cultivar (Chardonnay) and a drought-tolerant, indigenous cultivar (Xynisteri) were fully irrigated or drought-stressed and artificially inoculated with *P. viticola.* This obligate biotroph causes grapevine downy mildew, one of the most important diseases in European viticulture [33,34]. Interaction between both stresses is to be expected, since the stomata, the site of entry of *P. viticola*, are the plants’ first line of defense against drought stress. *P. viticola* can manipulate stomatal behavior [35] to facilitate infection and may thus potentially alter the drought response.

## 2. Results

### 2.1. Experimental Setup

**Figure 1:**
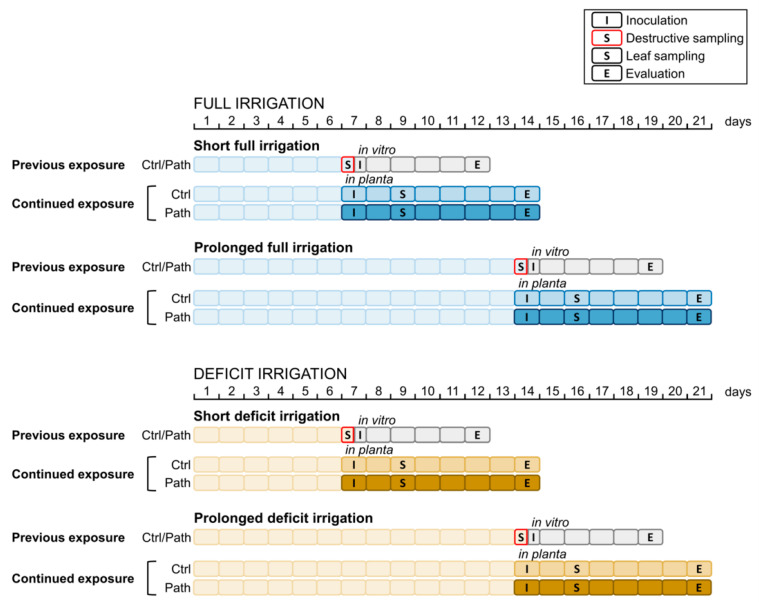
The 12 treatments used in this experimental setup. Each treatment group, represented by one timeline, consisted of five plants of both cultivars: drought-resistant Xynisteri and drought-sensitive Chardonnay. The plants were first exposed to 7 days of full/deficit irrigation (short full irrigation/short deficit irrigation) to establish the effect of short-term drought stress or to 14 days of full/deficit irrigation (prolonged full irrigation/prolonged deficit irrigation) to examine the effect of prolonged drought stress. At day 7 or 14, the plants were either sampled destructively or were maintained under the current irrigation regimen. Some of the sampled leaves of the first group were used for in vitro inoculation with water (Ctrl) or *Plasmopara viticola* (Path) to assess the effect of previous exposure to drought stress on disease development at 5 days post inoculation (dpi). The other groups were inoculated in planta, either with water (Ctrl) or with *P. viticola* (Path), to determine the effect of continued exposure to drought stress on disease development. These plants were maintained under full/deficit irrigation until disease evaluation at 7 dpi. From these plants, leaf samples were taken at 9 or 16 days of irrigation treatment (dot), corresponding to 1.5 dpi.

The experimental setup is provided in Figure 1. More details can be found in the Materials and Methods.

### 2.2. Basal Differences between Cultivars

**Figure 2:**
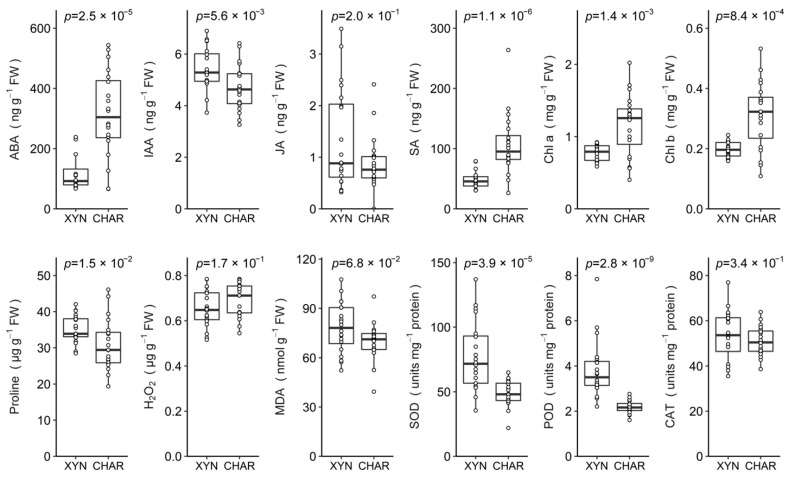
Basal levels of hormones, chlorophyll, and oxidative parameters in Xynisteri (XYN) and Chardonnay (CHAR). Samples from fully irrigated plants that were not inoculated with the pathogen were analyzed and pooled. The samples were collected from five plants of each cultivar at 7 and 14 days of full irrigation (previous exposure to full irrigation, before inoculation; see Figure 1) and at 9 and 16 days of full irrigation (control treatments of continued exposure to full irrigation; see Figure 1). The parameters are considered to be significantly different for both cultivars if the *p*-value is below 0.05 (Mann–Whitney *U*-test). All observations are shown as dots. The lines inside the box and the lower and upper boundaries of the box represent the median and first and third quartiles, respectively. The whiskers indicate the minimum and maximum values, excluding outliers. All observations outside the 1.5-fold interquartile range of the first or third quartile are considered outliers.

Figure 2 shows the basal levels of hormones, chlorophyll, and oxidative parameters in drought-tolerant Xynisteri and drought-sensitive Chardonnay plants without abiotic or biotic stress (control treatments for full irrigation; see Figure 1). Notably, in Chardonnay, abscisic acid (ABA) and salicylic acid (SA) levels were more than double, and the levels of chlorophyll a (Chl a) and chlorophyll b (Chl b) were almost double the levels in Xynisteri. In contrast, the peroxidase (POD) and superoxide dismutase (SOD) activities and the indole-3-acetic acid (IAA) and proline levels were significantly higher in Xynisteri than in Chardonnay. The malondialdehyde (MDA) content tended to be slightly higher in Xynisteri. No significant differences in H_2_O_2_ content, catalase (CAT) activity, and jasmonic acid (JA) content were observed between the cultivars.

### 2.3. Effect of Drought Stress on Disease Susceptibility

#### 2.3.1. Previous Exposure: In Vitro Inoculation

**Figure 3:**
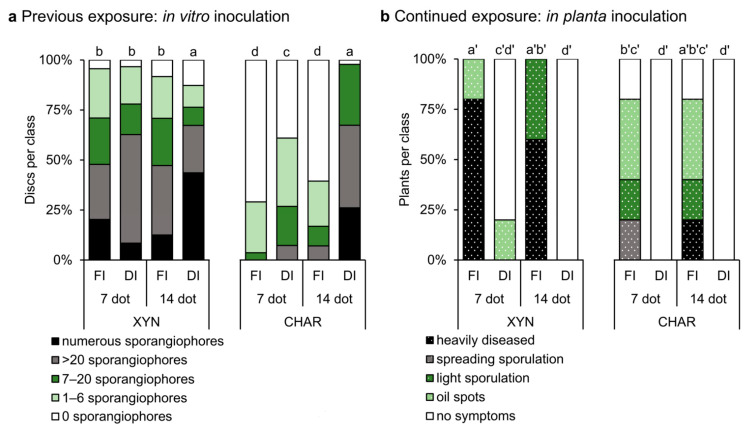
Influence of drought stress on the susceptibility of Xynisteri (XYN) and Chardonnay (CHAR) to *P. viticola*. The control plants were fully irrigated (FI), while the drought-stressed plants were exposed to deficit irrigation (DI). Different letters indicate significant differences (Mann–Whitney *U*-test; *p* = 0.05). (**a**) Previous exposure to drought stress: Plants were exposed to 7 or 14 dot before the leaves were detached (previous exposure to full/deficit irrigation; see Figure 1). Leaf discs were inoculated with a *P. viticola* sporangial suspension and each disc was evaluated 5 days later by counting the sporangiophores. Five plants of each cultivar were used per treatment, resulting in an average of 60 discs per treatment. Leaf discs from one plant of each cultivar, sampled at 14 days of deficit irrigation, could not be evaluated due to leaf scorch. (**b**) Continued exposure to drought stress: Plants were exposed to 7 or 14 dot prior to being sprayed with a *P. viticola* sporangial suspension (continued exposure to full/deficit irrigation; see Figure 1). The irrigation regimen was maintained until disease severity was evaluated 7 days later. Five plants of each cultivar were used per treatment.

To assess the effect of previous exposure to drought stress on the susceptibility to *P. viticola,* Xynisteri and Chardonnay plants were first exposed to full or deficit irrigation, for either 7 or 14 days of irrigation treatment (dot), before in vitro inoculation of leaf discs with the pathogen (previous exposure to full/deficit irrigation; see Figure 1). The disease evaluation results of the leaf discs are shown in Figure 3a. When watered sufficiently (full irrigation—FI), Xynisteri showed significantly higher disease severity than Chardonnay. On discs from non-stressed Chardonnay plants, almost no sporulation was observed. However, Chardonnay leaf discs became more susceptible to *P. viticola* when exposed to 7 days of deficit irrigation (DI). On Xynisteri leaf discs, *P. viticola* was able to grow quickly, irrespective of exposure to drought stress. As the duration of previous drought exposure increased from 7 to 14 days, the disease severity increased in both cultivars. In particular, in Chardonnay, longer exposure to drought stress drastically enhanced susceptibility, reaching a level similar to that of Xynisteri.

#### 2.3.2. Continued Exposure: In Planta Inoculation

In parallel, the effect of continued exposure to drought stress on the susceptibility to *P. viticola* was examined. Chardonnay and Xynisteri plants were subjected either to 7 or 14 days of full or deficit irrigation before being sprayed with a *P. viticola* sporangial suspension or distilled water (continued exposure to full/deficit irrigation; see Figure 1). The disease evaluation results of the plants are shown in Figure 3b. The irrigation regimen was maintained until the moment of disease evaluation, at 7 days post inoculation (dpi). Interestingly, under fully irrigated conditions, the indigenous cultivar Xynisteri showed more severe symptoms than the introduced cultivar Chardonnay. While the fully irrigated plants showed clear disease symptoms, the symptoms on plants challenged with a short drought stress period (7 days of deficit irrigation prior to inoculation) remained almost completely absent. Comparison of deficit-irrigated plants inoculated at 7 and 14 dot showed that the resistance to *P. viticola* did not significantly change with prolonged exposure to deficit irrigation before inoculation.

### 2.4. Field Measurements

**Figure 4:**
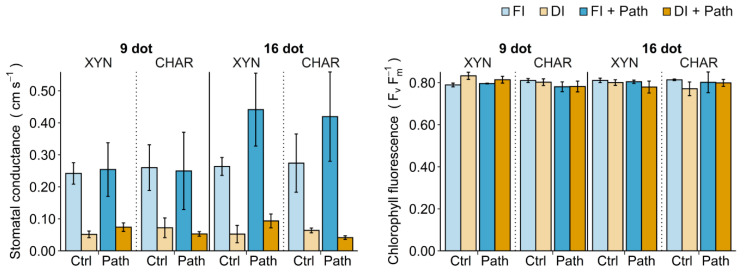
The impact of drought and pathogen stress on plant physiological parameters. Xynisteri (XYN) and Chardonnay (CHAR) plants were subjected to 7 or 14 days of full (FI) or deficit (DI) irrigation before in planta inoculation with water (Ctrl) or *P. viticola* (Path) (continued exposure to full/deficit irrigation; see Figure 1). Stomatal conductance and chlorophyll fluorescence were measured at 1.5 days post inoculation (dpi), corresponding to 9 or 16 days of irrigation treatment (dot). For the effect of drought stress, the fully irrigated and deficit-irrigated control plants should be compared. For the effect of pathogen stress, water-inoculated and pathogen-inoculated fully irrigated plants should be compared. The bars and error bars show the mean and the standard deviation, respectively.

Figure 4 shows how drought and pathogen stress affect stomatal conductance and chlorophyll fluorescence. The statistical analyses of the single-stress treatments are indicated in Table 1. The physiological parameters were not significantly different between plants with and without pathogens for both irrigation regimens (statistics not shown).

#### 2.4.1. Single Drought Stress

Drought stress had a profound effect on leaf stomatal conductance (Figure 4, ■ FI vs. ■ DI). After a short drought stress period (9 dot), the stomatal conductance had already plummeted in both cultivars. The chlorophyll fluorescence increased slightly in Xynisteri, but not in Chardonnay, after 9 days of deficit irrigation. Prolonged drought stress (16 dot) caused the chlorophyll fluorescence to decrease significantly in Chardonnay, but not in Xynisteri. 

#### 2.4.2. Single Pathogen Stress

Figure 4 shows how the stomatal conductance and chlorophyll fluorescence measurements changed in response to *P. viticola* inoculation on fully irrigated plants (Figure 4, ■ FI vs. ■ FI + Path). No significant differences in the physiological parameters were observed between plants inoculated with water and plants inoculated with *P. viticola* for the same irrigation regimen (Table 1). However, an increasing trend in stomatal conductance due to pathogen infection was observed at 16 dot.

#### 2.4.3. Combined Stress

When both stresses were combined (Figure 4, ■ DI + Path), the net effect on the physiological parameters was comparable to the strong effect of drought stress (Figure 4, ■ DI). The physiological parameters were not significantly different between drought-stressed plants with and without the pathogen. Xynisteri exhibited a slightly higher stomatal conductance in plants under combined stress than in plants under single drought stress. Conversely, a slightly lower stomatal conductance was found in Chardonnay under combined drought stress. 

### 2.5. Phytohormone Balance

**Figure 5:**
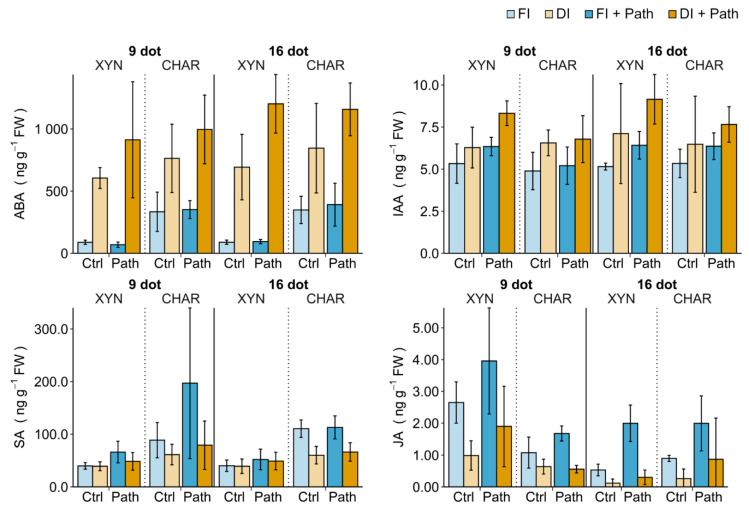
The impact of drought and pathogen stress on the leaf phytohormone balance. Xynisteri (XYN) and Chardonnay (CHAR) plants were subjected to 7 or 14 days of full (FI) or deficit (DI) irrigation before in planta inoculation with water (Ctrl) or *P. viticola* (Path) (continued exposure to full/deficit irrigation; see Figure 1). Samples for analysis were taken at 1.5 days post inoculation (dpi), corresponding to 9 or 16 days of irrigation treatment (dot). Each treatment consisted of five repetitions. For the effect of drought stress, the fully irrigated and deficit-irrigated control plants should be compared. For the effect of pathogen stress, water-inoculated and pathogen-inoculated fully irrigated plants should be compared. The bars and error bars show the mean and the standard deviation, respectively. Abbreviations: see Table 1.

Figure 5 shows how drought and pathogen stress affect the phytohormone content (continued exposure to full/deficit irrigation; see Figure 1). Statistical analysis of the single-stress treatments is provided in Table 1. Interactions among the cultivar, pathogen stress, and type and duration of irrigation were analyzed with a regression model (Appendix A). 

#### 2.5.1. Single Drought Stress

Short (9 dot) or prolonged (16 dot) deficit irrigation had a profound effect on the phytohormone balance (Figure 5, ■ FI vs. ■ DI). Drought stress caused significantly increased ABA and decreased JA levels in both cultivars, resulting in similar levels. Considering the large basal differences in ABA, Xynisteri produced much more ABA than Chardonnay in response to drought stress. Indeed, the ABA response to drought stress was likely cultivar-dependent (Appendix A Table A1). Moreover, ABA levels seemed to increase when drought stress was prolonged. Drought stress was also associated with a slight increase in IAA in both cultivars, which was only significant for Chardonnay at 9 dot. Finally, the SA response to drought stress was significantly dependent on the cultivar (Appendix A Table A1). Chardonnay responded to drought stress by decreasing its SA content, especially at 16 dot (Table 1), while still maintaining levels higher than those in Xynisteri. No clear SA response to drought was observed for Xynisteri (Figure 5).

#### 2.5.2. Single Pathogen Stress 

To assess the hormonal changes in the cultivars upon pathogen infection, plants were sprayed with water or *P. viticola* inoculum. Hormone analysis was performed on samples taken at 1.5 dpi from fully irrigated plants (Figure 5, ■ FI vs. ■ FI + Path). Independent repetitions were conducted at 9 and 16 dot, with only a slight change in plant age. Although Xynisteri was more susceptible to *P. viticola* than Chardonnay (Figure 3b), the hormonal responses of the cultivars to the pathogen were similar. Compared to water-sprayed plants, the pathogen-inoculated plants tended to accumulate more SA at 9 dot and JA at 16 dot (Figure 5). In the linear regression, there were indications that IAA was positively affected by the interaction with the pathogen, at least in Xynisteri (Appendix A Table A2). ABA levels remained unaffected by the pathogen (Figure 5).

#### 2.5.3. Combined Stress

The cultivars were subjected to 7 or 14 days of deficit irrigation before being sprayed with water or *P. viticola* inoculum to determine the combined effect of abiotic and biotic stress. The irrigation regimen was maintained and samples for analysis were taken at 1.5 dpi at 9 or 16 dot (Figure 5, ■ DI + Path). Interestingly, the ABA levels, which were already strongly increased by drought, increased even further 1.5 days after inoculation with the pathogen. The interaction between the pathogen and drought stress was highly significant for ABA (Appendix A Table A1). The additional ABA accumulation indicated that drought-stressed plants responded to the pathogen, although no symptoms were observed under drought stress (Figure 3b). Similarly, an increasing trend in IAA in response to the pathogen was observed in drought-stressed plants, already demonstrating increased levels of IAA due to drought. A significant interaction between drought and pathogen stress was also observed for JA (Appendix A Table A1). The accumulation of JA in response to the pathogen, which occurred in fully irrigated plants, was not apparent when deficit irrigation was applied (Figure 5). 

### 2.6. Chlorophylls and Oxidative Balance

**Figure 6:**
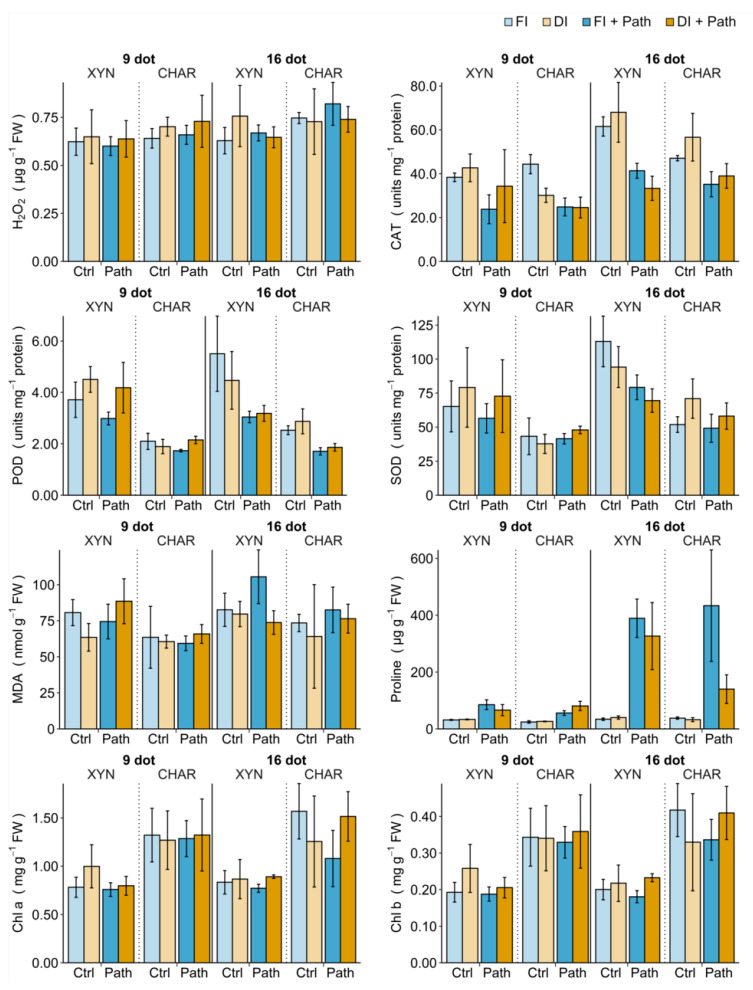
The impact of drought and pathogen stress on photosynthetic pigments and oxidative parameters. Xynisteri (XYN) and Chardonnay (CHAR) plants were subjected to 7 or 14 days of full (FI) or deficit (DI) irrigation before in planta inoculation with water (Ctrl) or *P. viticola* (Path) (continued exposure to full/deficit irrigation; see Figure 1). Samples for analysis were taken at 1.5 days post inoculation (dpi), corresponding to 9 or 16 days of irrigation treatment (dot). Each treatment consisted of five repetitions. For the effect of drought stress, the fully irrigated and deficit-irrigated control plants should be compared. For the effect of pathogen stress, water-inoculated and pathogen-inoculated fully irrigated plants should be compared. The bars and error bars show the mean and the standard deviation, respectively. Abbreviations: see Table 1.

Figure 6 demonstrates the impact of drought and pathogen stress on the chlorophyll content and oxidative parameters (continued exposure to full/deficit irrigation; see Figure 1). The statistical analysis of the single-stress treatments is indicated in Table 1. The interactions among cultivar, pathogen stress, and irrigation type and duration were analyzed with a regression model (Appendix A). 

#### 2.6.1. Single Drought Stress

To assess how short or prolonged drought stress affects the cultivars, plants were subjected to full or deficit irrigation for 9 or 16 days before samples were collected for analysis of the chlorophyll content and oxidative parameters (Figure 6, ■ FI vs. ■ DI). Even under drought stress, higher basal activities of SOD and POD in Xynisteri were maintained, just as the chlorophyll levels in Chardonnay remained significantly higher than those in Xynisteri.

Figure 6 shows differences in the responses of the cultivars to drought stress. In Xynisteri, the MDA content decreased significantly in response to a short drought stress (9 dot). Concurrently, the activities of the antioxidant enzymes (POD, SOD, and CAT) and the chlorophyll content tended to be elevated. With prolonged drought stress (16 dot), the activities of the antioxidant enzymes POD and SOD seemed to diminish and the H_2_O_2_ levels seemed to increase. Xynisteri’s response no longer included changes in the MDA or chlorophyll content. In Chardonnay, on the other hand, there was no apparent influence of a short drought stress (9 dot) on the MDA or chlorophyll content. The activity of the antioxidant enzyme CAT dropped significantly (Table 1). Continued drought stress (16 dot) eventually caused an increase in the activities of the antioxidant enzymes, significant for SOD (Table 1), while the chlorophyll content in Chardonnay seemed to decrease.

#### 2.6.2. Single Pathogen Stress

To determine how the cultivars responded to *P. viticola*, the plants were sprayed with water or pathogen inoculum. The samples for analysis were taken from fully irrigated plants at 1.5 dpi (Figure 6, ■ FI vs. ■ FI + Path). In this case, both time points (9 or 16 dot) could be seen as repetitions, with only a small change in plant age. Despite the difference in disease severity between the cultivars (Figure 3b), the responses of the cultivars to the pathogen were similar in terms of the parameters tested. What stood out most was the significant burst in proline associated with the pathogen-inoculated plants (Table 1). Additionally, the activities of the antioxidant enzymes (POD, SOD, and CAT) were lowered in response to the pathogen stress. These responses were most pronounced at 16 dot, coinciding with a significant increase in the MDA content in Xynisteri and a significant decrease in the Chl a level in Chardonnay (Figure 6). 

#### 2.6.3. Combined Stress

To examine the combined effect of both abiotic and biotic stress, the plants were subjected to 7 or 14 days of deficit irrigation before being sprayed with water or *P. viticola* inoculum. The irrigation regimen was maintained and samples for analysis were taken at 1.5 dpi at 9 or 16 dot (Figure 6, ■ DI + Path). Overall, the responses of deficit and fully irrigated plants to the pathogen were similar. The activities of the antioxidant enzymes CAT and POD were significantly reduced in pathogen-inoculated plants under full irrigation at 16 dot. Although no disease symptoms were seen on the drought-stressed plants (Figure 3b), a reduction was also observed in plants subjected to drought and pathogen stress. Similarly, a clear increase in proline in pathogen-inoculated plants was observed under both irrigation regimens. This indicates that proline and the activities of the antioxidant enzymes were associated with inoculation rather than disease incidence. 

The chlorophyll loss at 1.5 dpi in Chardonnay, on the other hand, might be associated with successful infection by *P. viticola*: in Chardonnay, the chlorophyll content seemed to increase with pathogen inoculation as drought was prolonged (16 dot), while it decreased under full irrigation (Figure 6). Indeed, for Chl a, the interaction between abiotic stress, its duration, and biotic stress was significant (Appendix A Table A1). MDA also seemed to be strongly associated with successful infection: the MDA content increased in fully irrigated Xynisteri at 16 dot upon inoculation with the pathogen. Deficit-irrigated, inoculated plants at 16 dot exhibited levels of MDA similar to those in the non-inoculated plants (Figure 6). The MDA response to the pathogen depended primarily on the interaction with the cultivar, drought stress, and its duration (Appendix A Table A1). 

### 2.7. Principal Component Analysis

**Figure 7:**
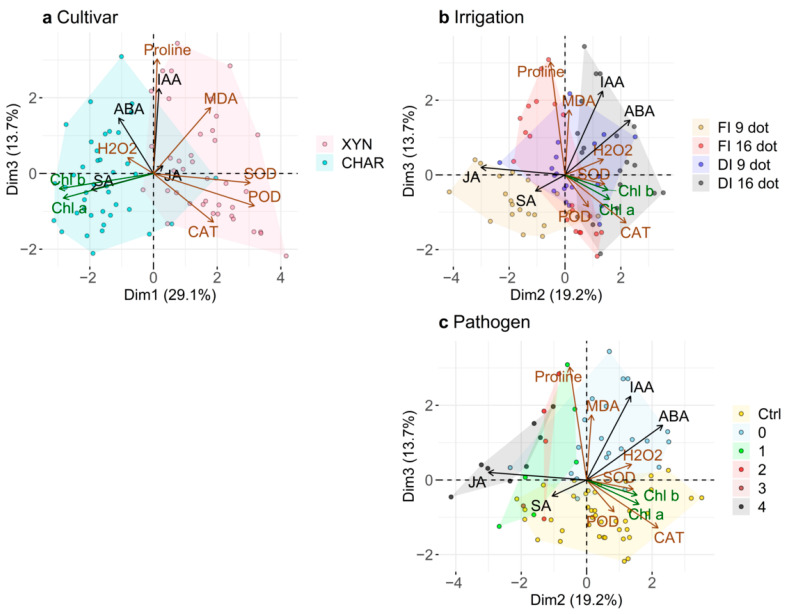
Principal component analysis visualizing the patterns in the phytohormone balance, oxidative balance, and chlorophyll content. Phytohormones are indicated with black arrows, oxidative parameters with brown arrows, and chlorophyll with green arrows. All plants (treated with continued exposure to full/deficit irrigation; see Figure 1) are represented by dots. Grouping is performed according to (**a**) cultivar: drought-tolerant Xynisteri (XYN) or drought-sensitive Chardonnay (CHAR); (**b**) drought stress: full (FI) or deficit (DI) irrigation and duration, at 9 or 16 days of irrigation treatment (dot); (**c**) pathogen stress: water-inoculated (Ctrl), no symptoms (0), oil spots (1), light sporulation (2), spreading sporulation (3), and heavily diseased (4). The first, second, and third principal components explained 29.1%, 19.2%, and 13.7% of the variation, respectively. Abbreviations: see Table 1.

Figure 7 shows the principal component analysis (PCA) results for the continued exposure to full/deficit irrigation (Figure 1). Although the cultivars and the irrigation and pathogen treatment were used as supplementary variables and did not participate in the construction of the dimensions, they allowed grouping of the data. A strong correlation was found between the first dimension (Dim1) and the cultivar (Table 2). Indeed, there was good separation between the two cultivars along the horizontal axis of the first dimension (Figure 7a). The association with the first dimension indicates that the differences between the cultivars, both at the basal level and in their response to the stresses, explained most of the variation in our dataset. Chardonnay was mostly associated with higher values for chlorophyll, SA, ABA, and H_2_O_2_, while Xynisteri was associated with higher POD, SOD, and CAT activities and MDA content (Table 3). The second dimension (Dim2) was mostly correlated with the irrigation regimen and, to a lesser extent, the duration of this treatment (Table 2). In the PCA, a shift is visible according to drought stress and its duration (Figure 7b). Drought-stressed plants were mostly associated with ABA, IAA, and CAT activity but were also positively correlated with the chlorophyll and H_2_O_2_ levels and SOD and POD activities (Table 3). Fully irrigated plants were mainly correlated with JA and with SA to a lesser extent (Table 3). Finally, the third dimension was primarily correlated with pathogen stress (Table 2). In Figure 7c, the water (Ctrl) and pathogen-inoculated plants showed clear separation along the vertical axis of the third dimension (Dim3), regardless of disease severity. Proline, IAA, and MDA seem to be strongly correlated and are associated with pathogen-inoculated plants.

Along the horizontal axis (Dim2) in Figure 7c, the plants are separated according to disease severity, with symptomless plants on the right and the most diseased plants on the left. Upon clustering according to drought stress in the same dimensions (Figure 7b), the group showing no symptoms overlapped with the drought-stressed group. Resistance was primarily observed in the 1st quadrant since a majority of the plants in the 3rd and 4th quadrants were not inoculated with the pathogen. The diseased plants were mainly clustered in the 2nd quadrant. The PCA suggests that JA is associated with infection under full irrigation. Interestingly, this PCA indicates that ABA and IAA play a role in drought-induced resistance, in which JA is no longer involved.

## 3. Discussion

### 3.1. Single Drought Stress

In this study, deficit irrigation in the absence of the pathogen led to changes in stomatal conductance and the phytohormone balance, but did not have prominent effects on oxidative parameters. For both cultivars, drought stress increasingly triggered ABA as the duration increased but negatively impacted JA. The SA content was only significantly affected in the drought-sensitive cultivar Chardonnay during prolonged drought stress. Although many studies have shown that JA and SA are involved in drought stress responses in addition to ABA [36], negative interactions of ABA with JA and SA have also been reported [37,38]. ABA can interfere with the JA-ethylene pathway in multiple ways [39], but whether the interaction is antagonistic [39] or synergistic [40] strongly depends on the conditions. A suppressive effect of ABA on the SA signaling pathway [41,42,43] has been shown for grapevine, particularly by Wang et al. [44] who showed that elicitation with exogenous ABA led to a gradual reduction in SA. 

It is to be expected that both cultivars have different mechanisms to cope with drought stress. The native cultivar Xynisteri is well-adapted to drought [45,46], while the drought-sensitive cultivar Chardonnay [32], which originates from French valleys with humid conditions, probably lacks adaptations to quickly cope with drought stress [45]. The higher basal activity of antioxidant enzymes (POD and SOD) and the higher basal levels of proline in Xynisteri may be part of its basal toolset to cope with oxidative stress. Plants generally respond to stresses with the production of reactive oxygen species (ROS), such as H_2_O_2_, as signaling molecules. This is typically followed by activation of the antioxidant system to limit the oxidative stress because ROS can severely damage many host cell components [47]. Proline has been associated with the detoxification of ROS in drought-stressed vines [48]. The higher basal IAA levels in Xynisteri may also contribute to its drought tolerance since IAA can help plants adjust their growth to unfavorable conditions through its crosstalk with ROS [49]. When Xynisteri was initially faced with drought (9 dot), chlorophyll fluorescence (a measure of the maximum photosystem II quantum efficiency) and chlorophyll levels boosted slightly and MDA levels reduced significantly compared to the fully irrigated control, suggesting Xynisteri might have developed mechanisms to avoid drought stress. In a drought-sensitive cultivar, the activation of the antioxidant system during drought might be weak or late [50]. Indeed, in Chardonnay, the activity of the antioxidant enzymes increased only during prolonged drought stress (16 dot). During the initial drought stress (9 dot), the CAT activity was even lowered in Chardonnay. Eventually, at 16 dot, the losses in chlorophyll fluorescence seemed higher in Chardonnay than in Xynisteri, suggesting that Chardonnay was more strongly affected by this level of drought.

### 3.2. Single Pathogen Stress

Weather conditions in Cyprus are extreme, with high light intensities and maximum daily temperatures reaching 45 °C in the shade. Previous studies have shown that both high temperature [51,52] and high light intensities [53] inhibit sporulation of *P. viticola*. Nevertheless, the pathogen was able to infect irrigated vines easily, probably because inoculations were performed in the evening and the conditions at night were optimal for infection, with minimum temperatures between 15 and 25 °C and relative humidity (RH) reaching 80 to 90%. 

Strikingly, both in vitro and in planta inoculations demonstrated that fully irrigated Xynisteri was more susceptible to the *P. viticola* isolate than fully irrigated Chardonnay, even though Chardonnay is generally considered to be susceptible to *P. viticola*. During this study, however, only limited sporulation was observed on fully irrigated Chardonnay plants, both in vitro and in planta. This is probably not due to the *P. viticola* FCH*Pv*1 isolate used for inoculation. In a phenotypic characterization of *P. viticola* isolates on a set of cultivars, *P. viticola* FCH*Pv*1 did not show deviant behavior and demonstrated virulence on cultivars carrying no major resistances to *P. viticola* [54]. It is more likely that even under full irrigation, the plants might have been experiencing additional abiotic stress due to the Cypriot climate. Basal levels of SA and ABA were high in fully irrigated Chardonnay, which may explain its more successful defense against *P. viticola* compared to Xynisteri. The lower activity of antioxidant enzymes could also be a part of the more successful pathogen defense strategy of Chardonnay since a sufficient oxidative burst can indeed restrain *P. viticola* [55,56]. The higher basal IAA and proline levels and higher antioxidant enzyme activity in Xynisteri, on the other hand, could be related to its high disease susceptibility under full irrigation. 

Infection by *P. viticola* at 1.5 dpi was associated with increased IAA, SA, and JA levels, strong decreases in antioxidant enzyme activity, and parallel bursts in proline, while stomatal conductance and chlorophyll fluorescence were only slightly affected. 

In contrast to drought stress, we found that pathogen stress acted positively on the JA and SA levels of both cultivars without an apparent effect on ABA levels. The increase in JA and SA at 1.5 dpi in infected, fully irrigated plants demonstrates that the plants were activating their defense mechanism. The roles of JA and SA have been extensively studied in resistant cultivars, in which both phytohormones accumulate at high levels after infection with *P. viticola* [57]. SA- as well as JA-mediated defense responses are implicated in the resistance to *P. viticola* [58,59,60,61,62]. Moreover, exogenous JA has been shown to protect grapevine leaf discs against *P. viticola* through callose deposition [63]. The dynamics of endogenous phytohormones during compatible interactions with *P. viticola* have, however, not been explicitly investigated. Polesani et al. [58] and Li et al. [62] observed increases in JA, coupled with very strong increases in methyl jasmonate (MeJA) during successful infection. The endogenous levels of both hormones increased from 12 to 48 h post inoculation but were eliminated once the tissue was completely invaded [58], indicating the involvement of JA and MeJA in the early developmental stages of the pathogen in compatible interactions. The PCA analysis in this study indicates that infection of fully irrigated plants was associated with elevated IAA. IAA levels during *P. viticola* infection have not been studied previously, so the origin and function of IAA accumulation still stand in question. Some pathogens are able to upregulate plant auxin signaling to suppress plant defenses, while others can synthesize IAA themselves through various pathways to increase pathogenesis [64]. 

It is well known that *P. viticola* can manipulate stomatal movements to facilitate infection [35]. In this study, the pathogen seemed to slightly increase stomatal conductance, particularly at 16 dot. This could be the result of IAA accumulation after infection [65], since the ABA levels were not substantially lowered in the presence of the pathogen. Stoll et al. [66] reported that stomatal conductance in irrigated plants decreased under infection with *P. viticola*, while other studies observed that the pathogen kept the stomata open by suppressing ABA production [67], by degrading ABA, or by blocking ABA transport [35]. *P. viticola* has also been shown to lower the photosynthetic rate [55,68] through the loss of chlorophyll; downregulation of the chlorophyll a/b binding protein, chlorophyll synthase, and Rubisco; and upregulation of chlorophyllase [69]. Chlorophyll losses recorded were still small at 1.5 dpi, likely because the chlorophyll content decreased only within the infected lesion [69,70] and might still have been insufficient to affect chlorophyll fluorescence [71].

The correlation between MDA, proline, and pathogen-inoculated plants in the PCA indicated that single pathogen stress caused lipid peroxidation and a burst in proline. Lipid peroxidation is highly correlated with the concentration of MDA, one of its final products. MDA enhances cell membrane damage, leading to cell death, and also acts as a signaling molecule. Lipid peroxidation is one of the most prominent symptoms of oxidative stress in animals and plants [72], but can also result from increased lipoxygenase activity. Lipoxygenases are associated with JA biosynthesis and involved in the activation of defense signaling against *P. viticola* [55]. Interestingly, the high accumulation of MDA, observed at 16 dot, was accompanied by strong decreases in antioxidative enzyme activity, although lipid peroxidation and a weak oxidative burst during the first 24 h of compatible infection with *P. viticola* have been associated with slight increases in total antioxidant capacity [55,56]. Inactivation of the antioxidant capacity to enhance pathogenesis-related (PR) gene expression [73,74] or to obtain stronger ROS production could be key in boosting plant defense and limiting pathogen infection. Despite the lowered activity of the antioxidant enzymes, however, H_2_O_2_ levels increased only slightly, which may be due to the proline bursts observed in pathogen-inoculated plants. Previous studies have also shown that proline accumulates under stress caused by *P. viticola* [75] and by drought [9,32,45,48]. Proline could have quenched and scavenged ROS, stabilizing proteins, DNA, and membranes [32,76]. Proline production might be part of the host defense mechanism against the oxidative stress caused in response to the pathogen, or part of the mechanism of the pathogen to impair the oxidative burst, restricting ROS to small concentrations that are insufficient to restrain the pathogen. 

### 3.3. Combined Stress

Because of the overlap and crosstalk between the responses to the individual stresses, the response to the concurrent pathogen and drought challenge could not be interpolated from the independent stress response. When drought and pathogen stress occurred simultaneously, the two stress responses interacted. The response to the pathogen seemed to interfere slightly with the adaptive strategies of Xynisteri to cope with drought stress, reducing the initial responses to drought in chlorophyll fluorescence and chlorophyll and MDA levels, and affecting the stomatal control. In plants under drought stress, pathogen inoculation at 1.5 dpi was associated with an additional increase in ABA. As expected, this resulted in a further decrease in stomatal conductance in Chardonnay. In contrast, in drought-stressed Xynisteri, the pathogen was associated with a slightly higher stomatal opening despite the pathogen-induced increase in ABA. In this cultivar, the combined stress also seemed to cause an additional increase in IAA, which has the ability to counteract ABA-induced closure [65].

Most interestingly, deficit irrigation induced resistance to *P. viticola* in both Chardonnay and Xynisteri. However, both in the susceptible fully irrigated and resistant deficit-irrigated plants, pathogen inoculation triggered losses of antioxidant enzyme activity and accumulation of proline, indicating that proline levels, in particular, can be a measure of pathogen stress whether the infection is successful or not. Under concurrent stress, the JA and SA levels were low and no longer substantially contributed to the pathogen defense response. Our results reveal that drought-induced ABA dominated the SA and JA defense responses occurring in response to *P. viticola* under full irrigation. Furthermore, a significant additional increase in ABA was observed in the inoculated compared to the non-inoculated drought-stressed plants, although infection under full irrigation did not trigger ABA. Thus, we hypothesize that ABA, rather than JA or SA, is involved in the observed drought-induced resistance to *P. viticola*. However, how might ABA contribute to the inhibition of infection by *P. viticola*? Considered a global regulator of plant stress responses, ABA is crucial in the response of plants to multiple stresses [14]. Its role in pathogen defense is poorly understood. Whether ABA acts as a positive or negative regulator of disease resistance is dependent on the stage of infection and the pathosystem but seems to be unrelated to the pathogen lifestyle or mode of attack [19]. Although most studies have established an antagonistic relationship between ABA and disease resistance [39,41,77,78,79], treatment of detached grapevine leaves with high concentrations of exogenous ABA has been shown to result in a reduction in *P. viticola* infections [63,80,81]. 

ABA can be involved in preinvasive defense, preventing pathogen penetration by controlling rapid stomatal movement [82]. Our data suggest, however, that the pathogen was not blocked completely during preinvasive defense. Fully and deficit-irrigated plants clearly differed in disease susceptibility, but both showed major changes in IAA and proline levels and antioxidant activities at 1.5 dpi. This indicates that the infection in the deficit-irrigated plants ceased post penetration and that *P. viticola* was able to penetrate the substomatal cavities, even though the stomatal conductance was markedly reduced. Notably, an additional increase in ABA was observed in deficit-irrigated plants after inoculation with the pathogen. This additional increase in ABA could be key to postinvasive resistance to this pathogen. During postinvasion defense, ABA is involved in callose [40,83] and stilbene [44] accumulation, thus limiting pathogen spread. ABA has also been found to accumulate strongly in some genetically resistant *Vitis* species after *P. viticola* inoculation [44,84]. In many resistant *Vitis* species, most infections never advance beyond the assessed developmental stage (24–48 hpi) [58,85].

In sharp contrast to the in planta drought-induced resistance, leaves from drought-stressed plants became more susceptible to the pathogen when inoculated in vitro. This indicates that drought-induced resistance depends on a rapid defense response, which can be reversed in a short time. The nature of this ABA-mediated defense remains to be investigated. We hypothesize that the recovered disease susceptibility in the detached leaves of drought-stressed plants is linked to their inability to maintain sufficiently high ABA levels once the drought stress is lifted and to restore the adverse effects of drought on pathogen defense in a timely manner. After all, drought severely interfered with the pathogen response by inducing IAA and antioxidant enzyme activity and antagonizing JA and SA levels. These adverse effects of drought on the pathogen response may have increased susceptibility to *P. viticola* in leaf discs. From this point of view, it is not surprising that post drought, Chardonnay partially lost its high tolerance to the pathogen. Previous exposure to deficit irrigation also deteriorated Xynisteri’s pathogen defense, but this cultivar was already extremely susceptible under full irrigation.

### 3.4. The Gap between In Vitro and in Planta Experiments

Depending on the inoculation occurring on leaf discs or intact plants, contradictory conclusions were reached regarding the impact of irrigation on susceptibility to *P. viticola*. Because of the perennial nature and size of the grapevine plant, many studies investigating the impact of compounds, microorganisms, resistance genes, or stress are performed on detached leaves. Understandably, the cutting itself, as well as the removal of the leaf from the elicitor of interest and the plant system, could trigger or inhibit responses in the leaf, resulting in responses different from those occurring in planta. In vitro studies of the plant response could oversimplify the system. This is especially the case when studying the effects of abiotic stress, since placing the leaves in controlled conditions partly relieves them from the abiotic stresses that the plants were experiencing. This study highlights the importance of being careful and critical in generalizing conclusions obtained through in vitro assays. In some cases, in vitro assays provide a good model, such as for the comparison of cultivar susceptibility under full irrigation. In other cases, extrapolation of the results of in vitro studies to the whole plant and field system proves impossible. Studies must take into consideration that a simplified model, such as the leaf disc assay, cannot be used without prior comparison with the whole-plant model. Similarly, we should bear in mind that results obtained with self-rooted cuttings may not reflect the stress responses of grafted vines in similar conditions [86,87]. 

### 3.5. Concluding Remarks

Climate change and the practices used to mitigate its effects may have a profound impact on plant pathogens. For sustainable vineyard management, the effect of deficit and full irrigation on the plant and its interaction with pathogens should be evaluated. Currently, deficit irrigation strategies in water-scarce regions are mainly studied for the effects on berry and wine quality, physiology, and yield [28,88,89]. In addition, deficit irrigation may help to avoid downy mildew epidemics. However, deficit irrigation might not be sufficient for introduced cultivars. For Chardonnay, irrigation is required for high yield and adequate grape quality in the Cypriot climate [90]. Because of the changing climate, it is increasingly important to choose plants that are well-suited to their planting location to minimize additional inputs. Future breeding programs in a context of climate change should pay special attention to the combination of biotic and abiotic stresses. The expected increase in abiotic stress might also be of importance in selecting resistance-inducing beneficial microorganisms or elicitors, since abiotic stress might interfere with the pathways needed to trigger resistance [31]. 

## 4. Materials and Methods

### 4.1. Site Description and Plant Material

This research was conducted in a sun-exposed area in Limassol, Cyprus (34°42’ N, 32°59’ E; elevation: 100 m a.s.l.), during 22 rainless days in May 2018. The climate is Mediterranean, with hot and dry summers. Supplementary Figure S1 presents the climatic data recorded with an on-site data logger (Kistock KH250; Kimo, Montpon, France). On an average day during the experiment, a maximum temperature of 38.3 °C was achieved in full shade, corresponding to 24% RH. On an average night, the minimum temperature dropped to 19.5 °C and the RH reached 70%.

This study included two cultivars of *Vitis vinifera*, namely Xynisteri and Chardonnay. Xynisteri is the main white grape cultivar grown in Cyprus, while Chardonnay is one of the most planted white grape cultivars worldwide. Chardonnay was introduced in Cyprus in the late 1980s. In 2014, Xynisteri and Chardonnay covered 30.2% and 1.6% of the circa 6142 ha viticultural area of Cyprus, respectively [8]. The clone of Chardonnay is unknown and, to our knowledge, no clones of Xynisteri have been identified [46]. Sixty self-rooted cuttings of each cultivar were planted in 8-litre polyethylene pots containing soil originating from the traditional vineyard area in Limassol. The soil properties were previously described by Tzortzakis et al. [45]. The soil had a clay-loam texture, an organic matter content of 2.19%, a total CaCO_3_ content of 66.9%, a pH of 7.42, and an electrical conductivity (EC) of 0.28 mS cm^−1^. The plants were grown in field conditions and automatically irrigated at field capacity using a drip irrigation system. Three months after planting, the plants were uniformly distributed into 12 treatment groups. The experimental setup is shown in Figure 1. For each treatment, five replicates were used per cultivar. Each group was treated with one of four abiotic stress treatments (7 or 14 days of full or deficit irrigation) to assess the effect of short and prolonged drought stress. Two groups were sampled destructively at 7 and 14 dot. In vitro inoculations were performed on discs of these leaves. In the evening, the remaining intact plants were inoculated with either the pathogen or water. For these plants, the irrigation regimen was maintained until disease evaluation 7 days later. Some leaves were sampled at 9 and 16 dot to establish the effect of pathogen attack at 1.5 dpi.

### 4.2. Abiotic Stress

Plants were either well-watered, in the full irrigation treatment, or exposed to drought stress by deficit irrigation. The fully irrigated plants received irrigation at field capacity from an automatic drip system every 6 h for 5 min. Deficit irrigation was maintained at 40% of the full irrigation based on the volumetric water content of the soil (VWC). To verify and accurately adjust the irrigation, the VWC was measured daily in about 8 randomly chosen pots using a portable time-domain reflectometer (TDR) (FieldScout TDR300 Soil Moisture Probe; Spectrum Technologies, Aurora, IL, USA) with 4.7-inch rods following watering of the full irrigation treatment (Supplementary Figure S2). The deficit-irrigated plants were irrigated manually approximately every two days according to the VWC. 

### 4.3. Biotic Stress

The effect of biotic stress and the combined effect of abiotic and biotic stress on the vine were examined by imposing biotic stress on the intact plants after 7 or 14 days of irrigation treatment (continued exposure to full/deficit irrigation; see Figure 1). To produce inoculum of the biotrophic pathogen *P. viticola*, the isolate FCH*Pv*1, obtained from Chardonnay in France, was grown for 10 days at 22 °C on detached Chardonnay leaves on water agar (0.65%). Sporangia were collected with distilled water and the suspension was adjusted to 2.5 × 10^4^ sporangia mL^−1^. Artificial inoculation was performed in the evening. The abaxial sides of all leaves were sprayed until run-off with a 3 mL sporangial suspension of *P. viticola*. The control plants were sprayed with distilled water. The irrigation regimens were maintained until disease evaluation. Since *P. viticola* needs 95–100% RH during the night for optimal infection and sporulation, each plant was equipped with a container of water and a moist plastic cover in the evening. To prevent extreme temperature development within the cover, the cover was removed in the morning and a light shade was created using a shadow mesh.

Sampling and disease evaluation were conducted at 1.5 and 7 dpi, respectively. Each plant was evaluated according to the following classification (based on the OIV descriptor 452 [91]): 0, no symptoms; 1, few oil spots with little to no sporulation; 2, moderate symptoms and non-spreading sporulation; 3, clearly diseased with spreading sporulation; and 4, severe symptoms with dense sporangiophore carpets.

### 4.4. Field Measurements

At 9 and 16 dot, stomatal conductance and chlorophyll fluorescence were recorded. The measurements were conducted on the 4th or 5th leaf starting from the apical meristem on randomly chosen plants at mid-morning, 4 h after onset of light. The stomatal conductance to water vapor was measured on three to five plants using a transient state diffusion porometer (AP4; Delta-T Devices, Cambridge, UK). The chlorophyll fluorescence (F_v_ F_m_^−1^), an indicator of the maximum quantum efficiency of photosystem II, was monitored on three or four plants after exposure to darkness for 20 min with a dark adaptation pin using a chlorophyll fluorometer (OS-30p; Opti-Sciences, Hudson, NH, USA). 

### 4.5. In Vitro Assessment of Disease Susceptibility

The 3rd and 4th leaves, counted from the apex, sampled at 7 and 14 dot (previous exposure to full irrigation; see Figure 1), were used to investigate the effect of the previous exposure to drought stress on the susceptibility to *P. viticola*. Leaf discs (11 mm diameter) were treated with 20 µL of distilled water or 20 µL of *P. viticola* sporangial suspension containing 2.5 × 10^4^ sporangia mL^−1^ and incubated on water agar (0.65%) at 22 °C (adapted from Schwander et al. [92]). At 5 dpi, the number of sporangiophores was counted to assign each disk to one of the following classes: 0, 0 sporangiophores; 1, 1–6 sporangiophores; 2, 7–20 sporangiophores; 3, more than 20 sporangiophores; and 4, numerous sporangiophores. An average of 60 discs was evaluated per treatment.

### 4.6. Quantification of Phytohormones

In leaves sampled at 7, 9, 14, and 16 dot, the levels of ABA, IAA, JA, and SA were determined, in ng per g fresh weight (FW). For each of the five replicates per treatment, two leaves were pooled, immediately frozen in liquid N_2_ and kept at −80 °C until analysis. The procedure for the quantification of phytohormones is described in detail by Haeck et al. [93]. The ground tissue (100 mg) was incubated with 5 mL of modified Bieleski extraction solvent (methanol/water/formic acid 75:20:5, *v*/*v*/*v*) for 20–24 h at −80 °C. After this cold extraction, filtration (30 kDa Amicon^®^ Ultra centrifugal filter unit, Merck Millipore, Overijse, Belgium), and evaporation (TurboVap^®^ LV, Biotage, Uppsala, Sweden), the extracts were reconstituted in 0.5 mL of methanol/water/formic acid (20:80:0.1, *v*/*v*/*v*). Chromatographic separation was performed on an ultra-high performance liquid chromatography system (UHPLC, Thermo Fisher Scientific, Erembodegem, Belgium) equipped with a Nucleodur C18 column (50 × 2 mm, 1.8 μm particle diameter, Macherey-Nagel, Düren, Germany). Mass spectrometric analysis was achieved in targeted single-ion monitoring mode on a Q-Exactive™ quadrupole-Orbitrap mass spectrometer (Thermo Fisher Scientific) equipped with a heated electrospray ionization source at a resolution of 70,000 full width at half maximum. In negative ionization mode, SA, ABA, and JA were measured using an elution gradient (300 µL min^−1^) of (A) methanol and (B) water, both with 0.01% formic acid. The formic acid concentration of solvent B was adjusted to 0.1% for the measurement of IAA in positive ionization mode. The following linear gradient was applied (solvent A): 0–1 min at 20%; 1–2.5 min from 20 to 45%; 2.5–9 min from 45 to 100%; 9–10 min at 100%; and 10–14 min at 20%. External and deuterated internal (d_4_-SA at 200 µg L^−1^, d_6_-ABA and d_5_-IAA at 1 µg L^−1^) standards were used for accurate quantification of the hormone content.

### 4.7. Quantification of Photosynthetic Pigments

Leaf samples were collected at 7, 9, 14, and 16 dot with five replications per treatment, each consisting of a pool of two leaves. The leaf tissue (100 mg) was incubated in a heat bath at 65 °C for 30 min with 10 mL of dimethyl sulfoxide (DMSO). The absorbance of the extract was measured at 645 and 663 nm using a microplate spectrophotometer (Multiskan GO; Thermo Fisher Scientific) and the Chl a and Chl b concentrations were calculated as described by Richardson et al. [94].

### 4.8. Quantification of the Hydrogen Peroxide Content, Lipid Peroxidation, and Proline Content

For quantification of the hydrogen peroxide (H_2_O_2_) content, lipid peroxidation (in terms of the MDA content), and the proline content, two leaves were sampled and pooled for each of the five plants per treatment at 7, 9, 14, and 16 dot. Fresh leaves were immediately frozen in liquid N_2_ and kept at −80 °C until analysis. Before analysis, the ground leaf tissue (200 mg) was homogenized with ice-cold 0.1% trichloroacetic acid (TCA). The extract was centrifuged and the supernatant was used for the quantification of H_2_O_2_ and MDA [95]. For quantification of H_2_O_2_, 0.5 mL of the supernatant was mixed with 0.5 mL of 10 mM potassium phosphate buffer (PPB) (pH 7.0) and 1 mL of 1 M potassium iodide (KI). The H_2_O_2_ content was calculated using standards of 5 to 500 μM H_2_O_2_ and a calibration curve was plotted accordingly. The absorbance was measured at 390 nm. For the MDA content, 0.5 mL of the supernatant was incubated with 1.5 mL of 0.5% thiobarbituric acid (TBA) in 20% TCA at 95 °C for 25 min. The reaction was stopped in an ice bath and the absorbance was measured at 532 and 600 nm. The MDA content was calculated using the extinction coefficient of 155 mM cm^−1^.

The proline content was also determined using the frozen ground tissue [96]. The leaf tissue (200 mg) was homogenized in 2 mL of 3% aqueous sulfosalicylic acid (SSA). The extracts were then centrifuged and 1 mL of the supernatant was incubated with 1 mL of acid ninhydrin and 1 mL of glacial acetic acid for 1 h at 100 °C. Then, the formed chromogen was extracted with toluene and the absorbance was measured at 520 nm using toluene as a blank. The proline concentration was determined using serial dilutions (0–100 μg mL^−1^) of D-proline. 

### 4.9. Quantification of Antioxidant Enzymes

The ground leaf samples were also used for determination of the activity of the antioxidant enzymes [97]. The tissue (200 mg) was homogenized with 3 mL of ice-cold 50 mM PPB (pH 7.0), including 1 mM ethylenediaminetetraacetic acid (EDTA), 1% *w/v* polyvinylpolypyrrolidone (PVPP), 1 mM phenylmethylsulfonyl fluoride (PMSF), and 0.05% polyethylene glycol tert-octylphenyl ether (Triton X-100). The homogenate was centrifuged at 16,000× *g* for 20 min at 4 °C. The supernatant was collected and an aliquot was first used to determine the protein content via the Bradford method [98], with bovine serum albumin (BSA) as the protein standard.

The CAT (EC 1.11.1.6) activity was determined by following the consumption of H_2_O_2_ (extinction coefficient 39.4 mM cm^−1^) at 240 nm for 3 min, as assayed by Jiang and Zhang [99]. The reaction mixture contained 100 mM PPB (pH 7.0), plant extract, and 200 μL of 75 mM H_2_O_2_. The results are expressed as CAT units per milligram of protein. One unit of enzyme decomposed 1 μmol of H_2_O_2_ per min.

SOD (EC 1.15.1.1) activity was assayed using a photochemical method [97]. The reaction mixture (1.5 mL) contained 50 mM PPB (pH 7.5), 13 mM methionine, 75 μM nitro blue tetrazolium (NBT), 0.1 mM EDTA, 2 μM riboflavin, and an enzyme aliquot. The reaction started after the addition of riboflavin. Tubes containing the reaction were then placed under a light source of two 15-Watt fluorescent lamps for 15 min. The reaction was stopped by placing the tubes in the dark. The reaction without the extract developed maximal color (control) and a nonirradiated mixture was used as a blank. The absorbance was determined at 560 nm and activity was expressed as SOD units per mg of protein. One unit of SOD activity was defined as the amount of enzyme required for 50% inhibition of the NBT photoreduction rate.

POD (EC 1.11.1.7) activity was determined according to the method used by Tarchoune et al. [100]. POD activity was assayed using pyrogallol, following the increase in absorbance at 430 nm, after oxidation to purpurogallin. The 2-mL reaction mixture contained 1665 μL of 100 mM PPB (pH 6.5), 200 μL of 100 mM pyrogallol, and 50 μL of extract. The reaction started with the addition of 85 μL of 40 mM H_2_O_2_. The increase in absorbance at 430 nm was measured on a kinetic cycle for 3 min. Calculations were performed using 2.47 mM cm^−1^ as the coefficient of extinction. One POD unit was defined as the amount of enzyme needed to decompose 1 μmol of H_2_O_2_ per min.

### 4.10. Statistical Analysis

All statistical analyses were conducted using R, version 3.6.1 [101]. The disease evaluation results were analyzed using the Kruskal–Wallis test, followed by the Mann–Whitney *U*-test (*p* = 0.05). For comparison of the other parameters between treatment groups, normality and homoscedasticity were first checked with Shapiro–Wilk’s and Levene’s tests (*p* = 0.05). Since the data did not meet the conditions of normality and homogeneity of variances, the results were analyzed using the Kruskal–Wallis test, followed by the Mann–Whitney *U*-test (*p* = 0.05). For analysis of the interactions among the cultivar, drought, and pathogen stress, a linear regression analysis was performed. A generalized least squares (GLS) model was improved by eliminating interaction terms until the lowest Akaike Information Criterion score (AIC) was reached.

## Figures and Tables

**Table 1:** *p*-values for the single drought stress and single pathogen stress. Significant differences (*p* ≤ 0.05) are marked with an asterisk. This analysis was performed on subsets of the data to exclude interactions. For the effect of single drought stress, fully irrigated and deficit-irrigated, water-inoculated plants were compared (control treatments of the continued exposure to full/deficit irrigation; see Figure 1). For the effect of single pathogen stress, water-inoculated and pathogen-inoculated fully irrigated plants were compared (continued exposure to full irrigation; see Figure 1).

	Effect of Drought Stress				Effect of Pathogen Stress			
	Xynisteri		Chardonnay		Xynisteri		Chardonnay	
Response Variables	9 dot	16 dot	9 dot	16 dot	9 dot	16 dot	9 dot	16 dot
Stomatal conductance	0.029 *	0.057	0.029 *	0.050 *	0.886	0.057	0.686	0.200
Chlorophyll fluorescence	0.100	0.663	0.400	0.029 *	0.164	0.657	0.268	1.000
Abscisic acid (ABA)	0.008 *	0.008 *	0.008 *	0.032 *	0.310	0.690	0.841	0.690
indole-3-acetic acid (IAA)	0.310	0.151	0.032 *	0.151	0.151	0.016 *	0.841	0.151
Jasmonic acid (JA)	0.008 *	0.008 *	0.095	0.012 *	0.222	0.008 *	0.095	0.056
Salicylic acid (SA)	1.000	0.841	0.151	0.008 *	0.032 *	0.548	0.056	1.000
Hydrogen peroxide (H_2_O_2_)	1.000	0.151	0.151	1.000	0.690	0.421	0.548	0.151
Catalase (CAT)	0.151	0.421	0.008 *	0.095	0.008 *	0.008 *	0.008 *	0.008 *
Peroxidase (POD)	0.056	0.690	0.310	0.310	0.151	0.008 *	0.151	0.008 *
Superoxide dismutase (SOD)	0.421	0.222	0.310	0.032 *	0.548	0.016 *	0.310	1.000
Malondialdehyde (MDA)	0.032 *	1.000	1.000	0.310	0.690	0.032 *	0.690	0.151
Proline	0.310	0.151	0.222	0.151	0.008 *	0.008 *	0.008 *	0.008 *
Chlorophyll a (Chl a)	0.095	0.841	1.000	0.421	1.000	0.222	0.841	0.032 *
Chlorophyll b (Chl b)	0.151	0.548	1.000	0.421	0.841	0.310	1.000	0.095

**Table 2:** Significant square correlation ratios (R^2^) of the supplementary variables in the principal component analysis (PCA). R^2^ indicates the strength of the correlation between the dimensions of the PCA and the variables cultivar (Chardonnay or Xynisteri), irrigation (full or deficit), duration (of the irrigation treatment; 9 or 16 dot), and pathogen (*P. viticola* or water inoculation). These supplementary variables were not involved in the construction of the dimensions. For each significant R^2^, the *p*-value is also shown.

Supplementary Variables	R^2^			*p*-Value		
	Dim1	Dim2	Dim3	Dim1	Dim2	Dim3
Cultivar	0.703			3.12 × 10^−22^		
Irrigation		0.355			5.40 × 10^−9^	
Duration		0.188	0.063		5.74 × 10^−5^	2.51 × 10^−2^
Pathogen		0.089	0.490		7.12 × 10^−3^	5.18 × 10^−13^

**Table 3:** Significant correlation coefficients of the active variables in the principal component analysis (PCA). The correlation coefficients describe the construction of the different dimensions of the PCA. For each significant coefficient, the *p*-value is also shown.

Active Variables	Correlation			*p*-Value		
	Dim1	Dim2	Dim3	Dim1	Dim2	Dim3
ABA	−0.292	0.624		8.61 × 10^−3^	6.21 × 10^−10^	
IAA		0.361	0.602		1.01 × 10^−3^	3.51 × 10^−9^
JA		−0.806			1.90 × 10^−19^	
SA	−0.523	−0.283		6.50 × 10^−7^	1.11 × 10^−2^	
H_2_O_2_		0.369			7.67 × 10^−4^	
CAT	0.505	0.586		1.75 × 10^−6^	1.14 × 10^−8^	
POD	0.850	0.225		1.88 × 10^−23^	4.48 × 10^−2^	
SOD	0.815	0.382		3.86 × 10^−20^	4.69 × 10^−4^	
MDA	0.481		0.467	6.24 × 10^−6^		1.25 × 10^−5^
Proline			0.812			5.85 × 10^−20^
Chl a	−0.759	0.428		3.32 × 10^−16^	7.37 × 10^−5^	
Chl b	−0.793	0.413		1.84 × 10^−18^	1.41 × 10^−4^	

## Data Availability

The data presented in this study are available in Supplementary Table S1.

## Supplementary Materials

The following are available online at https://www.mdpi.com/2223-7747/10/2/273/s1. Figure S1: Relative humidity (RH) and temperature during the experiment. The data were recorded every 20 min with an on-site data logger in sheltered conditions, Figure S2: Volumetric water content (VWC) of the soil during the experiment. Deficit irrigation (DI) was maintained at 40% of the full irrigation (FI) based on the volumetric soil water content. For both treatments, the VWC values shown were measured daily following watering of the FI treatment. The full line indicates the average VWC of about 8 pots at each time point. The bands illustrate the 95% confidence intervals, Table S1: Main data supporting the results of this article.

## Appendix A

**Table A1:** *p*-values for the different factors and interactions retained in the models after model selection. Interaction terms that were not important for the models were removed based on the AIC. The linear regression analyzes the interactions between the predictor variables: cultivar (Chardonnay or Xynisteri), irrigation (full or deficit), duration (of the irrigation treatment; 9 or 16 dot) and pathogen (*P. viticola* or water inoculation). Values are marked with a single asterisk if *p*-value <10% and with a double asterisk if *p*-value < 5%.

Predictor Variables	ABA	IAA	JA	SA	H_2_O_2_	CAT	POD	SOD	MDA	Pro	Chl a	Chl b
Cultivar	0.3257	0.7236	0.2221	0.3798	0.9512	0.0251 **	0.0106 **	0.0007 **	0.0972 *	0.0658 *	0.0028 **	0.0011 **
Irrigation	0.0000 **	0.0000 **	0.0000 **	0.0118 **	0.9476	0.0047 **	0.1392	0.0333 **	0.3146	0.1133	0.0279 **	0.0277 **
Duration	0.1155	0.1011	0.0596 *	0.8415	0.3598	0.0000 **	0.0000 **	0.0001 **	0.7006	0.0350 **	0.4966	0.3729
Pathogen	0.0002 **	0.1101	0.1496	0.8354	0.6716	0.0000 **	0.0000 **	0.0726 *	0.1867	0.0103 **	0.0470 **	0.0226 **
Cult:Irrig	0.0514 *		0.2309	0.0039 **	0.0868 *	0.1119	0.1119	0.0028 **	0.6224	0.0221 **	0.0457 **	0.0356 **
Cult:Durat			0.0798 *	0.6964	0.5036	0.8031	0.1401	0.0871 *	0.3390	0.9134		
Irrig:Durat	0.1166		0.5044	0.6556	0.4346	0.0001 **	0.0709 *	0.1419	0.6275	0.0529 *	0.1446	0.1040
Cult:Pathog		0.1325	0.4623	0.7989	0.1622	0.0080 **	0.6546	0.0123 **	0.1679	0.0027 **	0.1097	0.0956 *
Irrig:Pathog	0.0002 **		0.0105 **	0.9742	0.0760 *	0.0323 **	0.4419		0.8025	0.0004 **	0.0008 **	0.0035 **
Durat:Pathog	0.1203		0.1078	0.3247	0.3329	0.0331 **	0.0000 **	0.0114 **	0.5933	0.2050	0.1565	0.1146
Cult:Irrig:Durat			0.0033 **		0.1068	0.0012 **	0.0158 **	0.0014 **	0.2692	0.0343 **		
Cult:Irrig:Pathog			0.1453				0.1241		0.0876 *	0.0486 **	0.0331 **	0.0741 *
Cult:Durat:Pathog			0.1684	0.1618		0.0404 **	0.7027		0.0442 **	0.0011 **		
Irrigat:Durat:Pathog				0.1417	0.1659	0.0030 **	0.0141 **		0.7374	0.0002 **	0.0337 **	0.0687 *
Cult:Irrig:Durat:Pathog							0.1503		0.0438 **	0.0197 **		

**Table A2:** Coefficient estimates for the different factors and interactions retained in the models after model selection. Interaction terms that were not important for the models were removed based on the AIC. The linear regression analyzes the interactions between the predictor variables: cultivar (Chardonnay or Xynisteri), irrigation (full or deficit), duration (of the irrigation treatment; 9 or 16 dot) and pathogen (*P. viticola* or water inoculation). Coefficients are marked with a single asterisk if *p*-value < 15% and with a double asterisk if *p*-value < 5%.

Predictor Variables	ABA	IAA	JA	SA	H_2_O_2_	CAT	POD	SOD	MDA	Pro	Chl a	Chl b
Xynisteri	−87.40	0.15	−0.15	−18.11	0.00	9.20 **	1.60 **	25.57 **	15.56 *	7.48 *	−0.33 **	−0.10 **
Full	−483.00 **	−1.66 **	0.63 **	53.06 **	0.00	−11.74 **	−0.35 *	−14.03 **	9.37	5.44 *	0.29 **	0.08 **
9 dot	−141.10 *	−0.50 *	0.36 *	−4.51	−0.05	−26.61 **	−0.98 **	−30.10 **	−3.57	−6.21 **	0.07	0.03
Pathogen	349.77 **	0.68 *	0.53 *	4.68	−0.02	−19.84 **	−1.01 **	−10.20 *	12.33	107.46 **	0.26 **	0.08 **
Xynist:Full	−183.70 *		−0.21	−54.85 **	−0.10 *	7.46*	1.38 *	28.35 **	−6.47	−11.24 **	−0.31 **	−0.09 **
Xynist:9 dot			0.56 *	10.15	−0.04	1.42	1.01 *	15.85 *	−12.59	−0.45		
Full:9 dot	140.71 *		−0.17	−11.61	−0.06	24.01 **	0.55 *	13.57 *	−6.37	−7.35 *	−0.22 *	−0.07 *
Xynist:Pathog		0.91 *	−0.31	6.63	−0.06	−12.66 **	−0.28	−16.60 **	−18.23	179.03 **	−0.24 *	−0.07 *
Full:Pathogen	−343.30 **		0.65 **	−0.84	0.11 *	10.13 **	0.19		−3.28	288.00 **	−0.65 **	−0.15 **
9 dot:Pathogen	−25.24 *		−0.59 *	31.49	0.06	12.35 **	1.26 **	16.80 **	−7.02	−53.04	−0.22	−0.06 *
Xynist:Full:9 dot			1.31 **		0.14 *	−22.13 **	−2.37 **	−43.01 **	20.60	11.40 **		
Xynist:Full:Pathog			0.55 *				−1.37 *		32.07 *	−219.50 **	0.46 **	0.10 *
Xynist:9 dot:Pathog			0.88	−51.84		13.70 **	−0.29		37.94 **	−200.70 **		
Full:9 dot:Pathog				54.49 *	−0.12	−20.20 **	−0.80 **		−6.23	−311.00 **	0.46 **	0.11 *
Xynist:Full:9 dot:Pathog							1.57		−53.75 **	263.41 **		
