# Supplementary figures and images for: Responses to Drought Stress Modulate the Susceptibility to Plasmopara viticola in Vitis vinifera Self-Rooted Cuttings
Source: Plants (Basel). 2021 Jan 30;10(2):273. doi: 10.3390/plants10020273 (PMC7912678; doi:10.3390/plants10020273)

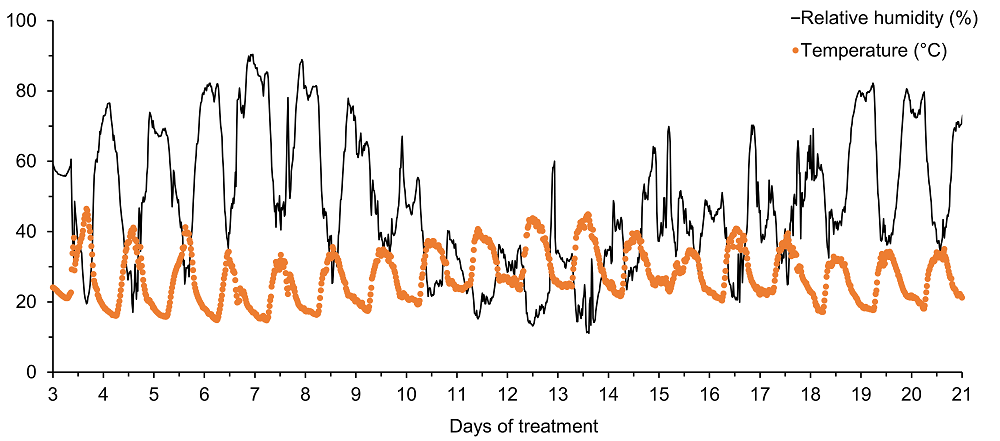

Supplement: Supplementary file 1 [file plants-10-00273-s001.zip › Figure S1 (1).tif]

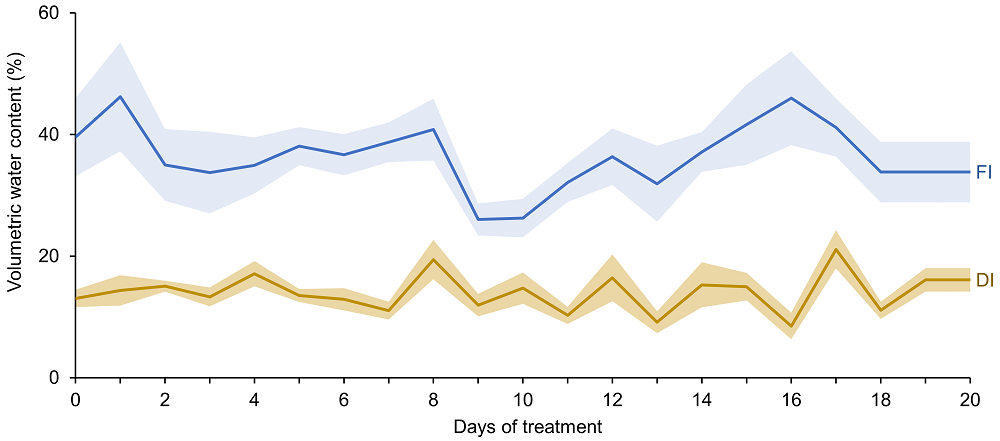

Supplement: Supplementary file 1 [file plants-10-00273-s001.zip › Figure S2 (1).tif]
